# Delivering trauma and rehabilitation interventions to women and children in conflict settings: a systematic review

**DOI:** 10.1136/bmjgh-2019-001980

**Published:** 2020-04-23

**Authors:** Reena P Jain, Sarah Meteke, Michelle F Gaffey, Mahdis Kamali, Mariella Munyuzangabo, Daina Als, Shailja Shah, Fahad J Siddiqui, Amruta Radhakrishnan, Anushka Ataullahjan, Zulfiqar A Bhutta

**Affiliations:** 1 Centre for Global Child Health, Hospital for Sick Children, Toronto, Ontario, Canada; 2 Health System and Services Research, Duke-NUS Medical School, Singapore; 3 Center of Excellence in Women and Child Health, Aga Khan University, Karachi, Pakistan

**Keywords:** traumatology, surgery, health services research, public health, systematic review

## Abstract

**Background:**

In recent years, more than 120 million people each year have needed urgent humanitarian assistance and protection. Armed conflict has profoundly negative consequences in communities. Destruction of civilian infrastructure impacts access to basic health services and complicates widespread emergency responses. The number of conflicts occurring is increasing, lasting longer and affecting more people today than a decade ago. The number of children living in conflict zones has been steadily increasing since the year 2000, increasing the need for health services and resources. This review systematically synthesised the indexed and grey literature reporting on the delivery of trauma and rehabilitation interventions for conflict-affected populations.

**Methods:**

A systematic search of literature published from 1 January 1990 to 31 March 2018 was conducted across several databases. Eligible publications reported on women and children in low and middle-income countries. Included publications provided information on the delivery of interventions for trauma, sustained injuries or rehabilitation in conflict-affected populations.

**Results:**

A total of 81 publications met the inclusion criteria, and were included in our review. Nearly all of the included publications were observational in nature, employing retrospective chart reviews of surgical procedures delivered in a hospital setting to conflict-affected individuals. The majority of publications reported injuries due to explosive devices and remnants of war. Injuries requiring orthopaedic/reconstructive surgeries were the most commonly reported interventions. Barriers to health services centred on the distance and availability from the site of injury to health facilities.

**Conclusions:**

Traumatic injuries require an array of medical and surgical interventions, and their effective treatment largely depends on prompt and timely management and referral, with appropriate rehabilitation services and post-treatment follow-up. Further work to evaluate intervention delivery in this domain is needed, particularly among children given their specialised needs, and in different population displacement contexts.

**PROSPERO registration number:**

CRD42019125221.

**Key questions**

**What is already known?**

- Armed conflict causes direct harm to civilians and has detrimental consequences for health systems, with impacts on infrastructure and health facilities affecting the provision of and access to health service including trauma and rehabilitation care.
- Acts of violence in the context of contemporary armed conflict are increasingly occurring in urban areas among civilian populations, with implications for the kinds of injuries sustained and the types of trauma and rehabilitation services needed at the population level.
- Traumatic injury from exposure to violence, land mines and other explosive remnants of war can cause devastating and long-lasting physical and psychological disability. Prompt management of traumatic injuries, and long-term follow-up care and rehabilitative services are vital to improve quality of life.

**What are the new findings?**

- Very little of the existing literature on the delivery of trauma and rehabilitation interventions to conflict-affected women and children focuses on the delivery of interventions targeted at women or children specifically, and no intervention effectiveness evidence for either of these populations is reported.
- Most of the literature on the delivery of trauma and rehabilitation interventions to conflict-affected women and children documents the use of highly skilled or specialised health staff providing surgical management in inpatient or fixed outpatient facilities, but the delivery of a range of interventions through mobile or outreach modes was also documented. The availability (or lack) of specialised expertise for treating paediatric patients, and of medical supplies and surgical equipment appropriate for paediatric patients, was reported as an important factor affecting intervention delivery.
- There is very little documentation in the literature of the delivery of relevant community-based interventions, with a single publication reporting on a mine risk reduction programme delivered by community health workers, teachers and civic leaders.

**Key questions**

**What do the new findings imply?**

- As the number of women and children affected by armed conflict continues to increase, the lack of evidence on effective approaches for reaching these populations with appropriate trauma and rehabilitation services remains a significant knowledge gap.
- Further work is needed to evaluate trauma and rehabilitation intervention effectiveness among women and children and to identify effective intervention delivery approaches for these populations in different population displacement contexts.

## Background

As the nature of conflict is changing, acts of violence in the context of contemporary armed conflict are increasingly occurring in urban areas among civilian populations leading to deaths and life-changing injuries as a result of trauma.^1–3^ In 2017, 92% of civilian deaths and injuries by explosive weapons were reported to occur in populated areas.^4^ Children suffer immensely from the consequences of these brutal trends, and almost one-fifth of children worldwide are now living in areas affected by armed conflict.^2 5^ The *Landmine Monitor*, an initiative that provides systematic monitoring and assessment of the international community’s response to the issues caused by land mines, cluster munitions and other explosive remnants of war (ERW), reported that in 2017, children accounted for 47% of all civilian casualties where the age was known, and women and girls made up 13% of all casualties where the sex was known.^6^ Even long after a conflict has ended, children are particularly vulnerable to injuries from land mines and ERWs, often mistaking them for toys, or picking up scrap metal to sell for cash.^7 8^

Physical trauma in any setting often occurs suddenly, causing life-threating and life-altering injuries which can require extensive measures for survival and a range of subsequent rehabilitation services aimed at improving quality of life, sustaining and maintaining functionality and developing adaptive capacity. In development settings in low and middle-income countries (LMIC), trauma and rehabilitation capacity are often already highly deficient,^9 10^ rendering an effective response to the onset and continuation of humanitarian crises extremely challenging. Armed conflict causes direct harm and injuries to civilians, and it has profound negative impacts on health systems and health-supporting infrastructure such as electricity and transportation, and jeopardises health facility supply chains, creating shortages of medicine and medical supplies.^11^ Conflict affects the delivery of services to those in need by increasing both the demand for interventions and the difficulties in accessing those interventions.^11^

Armed conflict provokes a wide variety of injuries, and while everyday trauma still persists, conflict-specific injuries differ from those seen in everyday trauma practice.^12^ Blunt injuries from collapsing buildings, penetrating wounds caused by high-velocity projectiles, explosive blast injuries and extensive burn trauma require technical expertise in resuscitation, critical care, care of burn wounds and the treatment of complex abdominal, vascular and orthopaedic injuries.^13 14^ Management of acute trauma and injuries in conflict zones most often require a prompt response to deliver immediate medical attention and mobile care. Patients’ survival often depends on the length of time from the point of injury to a health facility with surgical capacity. The Sphere Handbook, a key resource for humanitarians, outlines minimum standards of humanitarian response for multiple sectors, including health.^15^ To reduce the impact of injuries and the risk of health system collapse, Sphere recommends providing systematic triage and mass casualty management alongside basic emergency, safe operative and rehabilitative care. Ensuring standardised protocols exist to include acuity-based triage, front-line emergency care and referrals for emergency and advanced care, and those responding to mass casualties are adequately trained and skilled. Prehospital care allows for timely provision of life-saving measures such as first aid, resuscitation, proper wound and burn care and adequate immobilisation of fractures. First aid provided at the community level by trained volunteers and non-medical professionals helps ensure that victims receive life-sustaining care promptly.^15^

A previous systematic review examined the quantity and quality of the literature on the provision of injury and rehabilitation services in humanitarian crises. Sixty-two per cent of the reviewed studies were conducted in conflict settings or with patients who had suffered conflict-related injuries, and 98% of the papers described interventions for the general population.^16 17^ The authors recommended that although there is some documented evidence on the effectiveness of injury and rehabilitation interventions in humanitarian settings, more research is needed on the most effective ways of delivering health interventions.^16^

This current review is one of a set of eight systematic reviews examining the delivery of health and nutrition interventions to conflict-affected women and children. Here we aimed to systematically synthesise the indexed and grey literature reporting specifically on the delivery of trauma and rehabilitation interventions to women and children in conflict settings, to identify how, where and by whom these interventions have been and are being delivered and to better understand existing intervention gaps in this domain. We ultimately aim for this review to help inform future implementation and delivery guidance in this domain.

## Methods

### Literature search

A systematic search of literature published from 1 January 1990 to 31 March 2018 was conducted in MEDLINE, Embase, CINAHL and PsycINFO using Medical Subject Headings terms and keywords related to three concepts: (A) conflict; (B) women and children; and (C) trauma and rehabilitation. Conflict-related search terms included war, crisis, refugees, internally displaced person (IDP) and stateless. Population-related words included women, children, pregnant, adolescents and newborn. Terms related to trauma and rehabilitation included war-related trauma, amputation, traumatic brain injury, orthopaedics, physiotherapy, and so on. The complete search syntax used for the MEDLINE database is included in online supplementary appendix B. We also screened reference lists of key systematic reviews conducted previously in the field of humanitarian health.

For grey literature, we searched the websites of 10 major humanitarian agencies and organisations that are actively involved in humanitarian health research or response for women and children in conflict situations for reports on the implementation of health interventions in our population of interest. These websites included: Emergency Nutrition Network, International Committee of the Red Cross, International Rescue Committee, Médecins Sans Frontières, Save the Children, United Nations Population Fund, United Nations High Commissioner for Refugees, UNICEF, Women’s Refugee Commission and World Vision. We used broad terms for conflict and health interventions tailored to the search functionality of each website. Documents published from 1 January 2013 to 30 November 2018 were reviewed.

### Eligibility criteria

Eligible publications were those reporting on populations affected by conflict in low or middle-income countries, as classified by the World Bank^18^ and describing the delivery of a trauma or rehabilitation intervention during or within 5 years of the cessation of a conflict. The intervention was required to target or include neonates, children, adolescents or women of reproductive age, and injuries that were conflict related. In order to identify the most informative resources from the large volume of grey literature available, eligible grey literature publications were additionally required to explicitly report on the delivery site and the delivery personnel for each intervention.

Non-English publications; case reports of single patients; publications reporting on military personnel, refugee populations bound for a high-income country, economic or mathematical modelling; and editorials and opinion pieces were excluded from our review. Other excluded publications included systematic reviews, guidelines and publications where no specific health intervention was described (eg, prevalence studies).

### Data extraction and analysis

All identified indexed records were downloaded into EndNote and duplicates were removed. Unique records were subsequently imported into Covidence for screening. Titles and abstracts were reviewed in duplicate, and the full text of each potentially relevant publication was then screened by one reviewer, with reasons for exclusion noted at this stage.

Data and information from the indexed and grey literature publications that met the review eligibility criteria were extracted in duplicate into a customised Research Electronic Data Capture form. Key variables with regard to authors and publication year, setting, target population, study design and intervention descriptions were extracted. The double-entered data were matched and any inconsistencies identified were corrected.

Descriptive statistics were generated to summarise key characteristics of the population and intervention, including population displacement status and intervention delivery characteristics, and we narratively synthesised reported information on the factors impeding or facilitating intervention delivery. We tabulated any reported data on intervention coverage and effectiveness measured in relation to our population of interest; given the scarcity of such data, no meta-analysis was attempted.

### Patient and public involvement

Neither patients nor the public were involved in the conduct of this systematic review.

## Results

**Figure 1:**
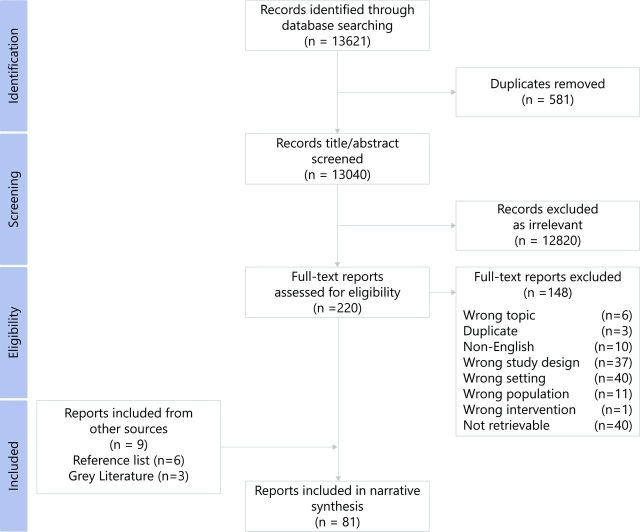
Preferred Reporting Items for Systematic Reviews and Meta-Analyses (PRISMA) flow diagram: publication selection process for systematic review on the delivery of trauma and rehabilitation interventions to women and children in conflict settings.

**Figure 2:**
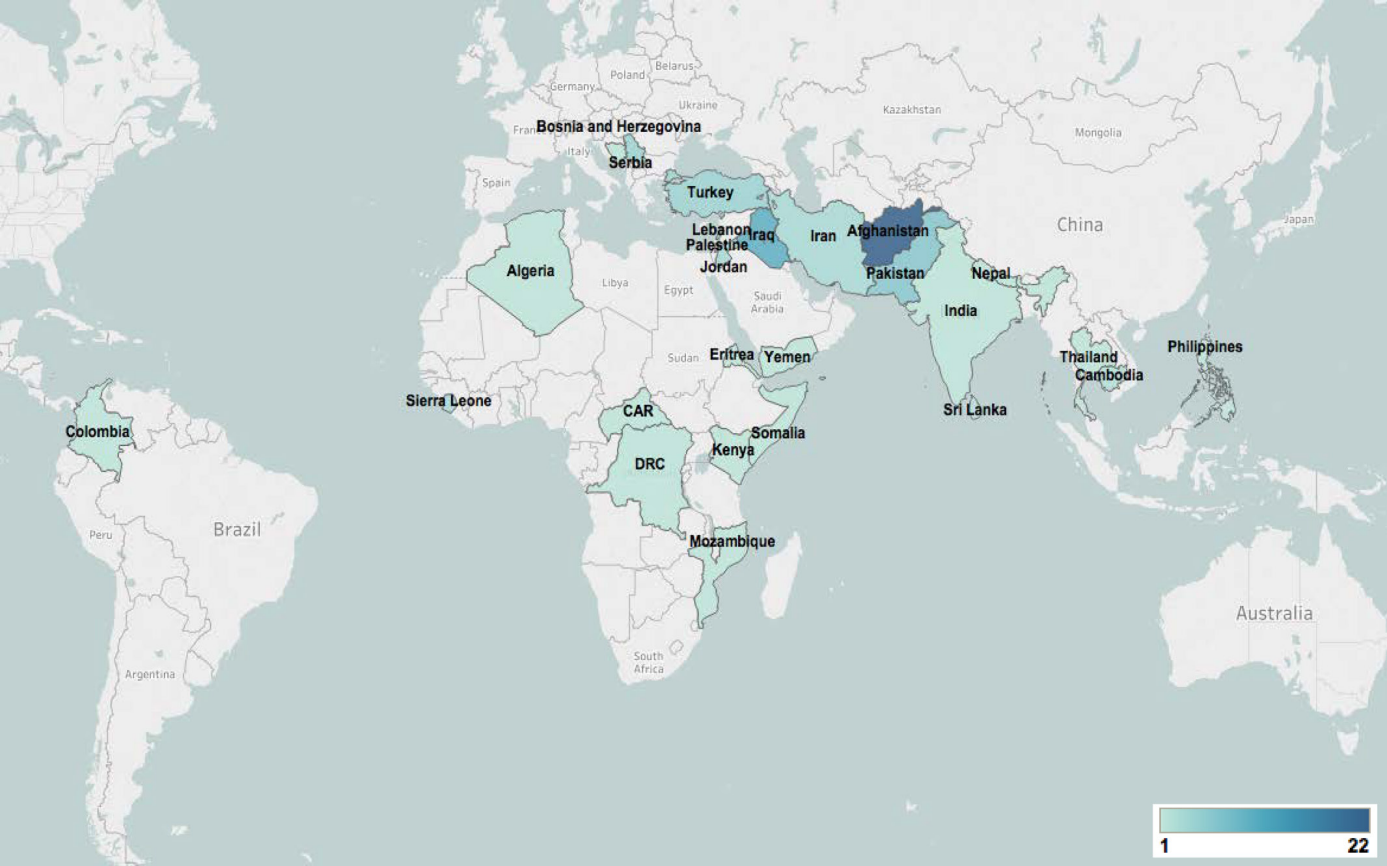
Geographic distribution of included publications.

We identified 13 040 unique citations from indexed databases, 220 of which were screened as potentially relevant and assessed in full text, with 72 meeting our inclusion criteria (figure 1). Six additional publications identified from the reference list of a previous systematic review and three grey literature publications also met our eligibility criteria. A total of 81 publications^19–99^ reporting on interventions delivered in 26 countries were ultimately included in our review (figure 2). Delivery characteristics of the included studies are provided in online supplementary appendix A. Notably, more than a quarter of all included publications focused on intervention delivery in Afghanistan (22/81; 27%), with Iraq being the next most frequently reported country (10/81; 12%).

**Figure 3:**
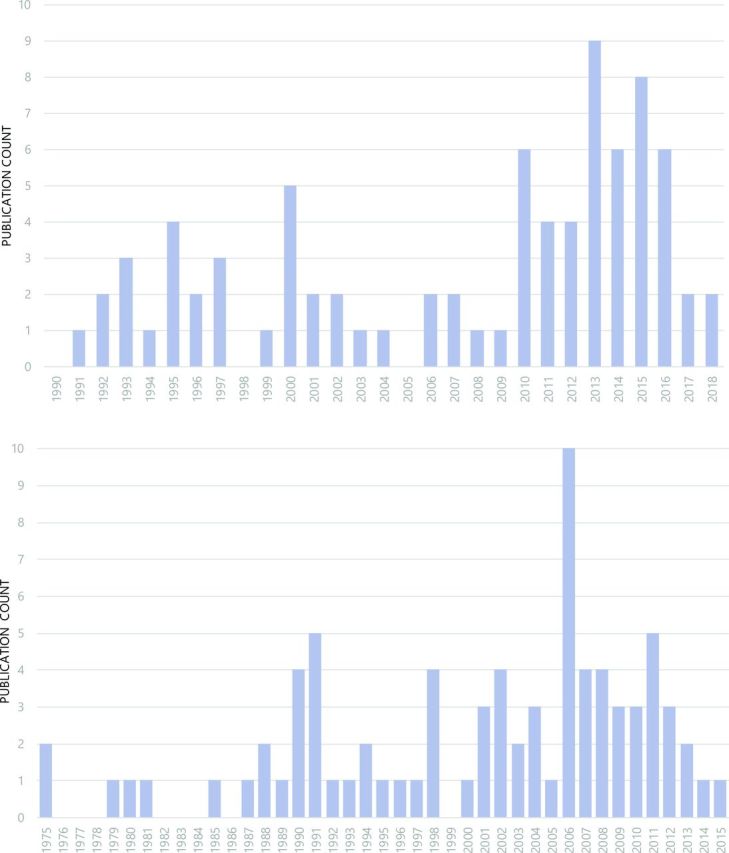
Publications counts by publication year (top) and intervention start year (bottom).

Publications ranged from January 1991 to March 2018. Despite yearly variations in the frequency of publications by year, these numbers remained low. The greatest number of publications from a single year was seen in 2013, with a total of 9. The number of publications shows a peak in 2010 and again in 2013. No eligible publications were identified during 1990, 1998 and 2005. The start years of interventions reported spanned from 1975 to 2015, with peaks in 1990-91 and 2006, and remained relatively high in comparison to the previous years (figure 3).

Of the included publications, 16% (13/81) reported the delivery of interventions specifically targeted at child and adolescents, but no publications reported on interventions specifically targeting women. The majority of publications (84%, 68/81) reported interventions that included the general population. In regard to displacement status, half of the publications (49%, 40/81) reported on intervention delivery in conflict-affected populations that were not displaced, 6% (7/81) reported on intervention delivery in refugee populations and 4% (4/81) in internally displaced populations (IDP). The existing healthcare system and military-based healthcare were the most commonly reported platforms involved in the delivery of interventions, reported by 49% and 33% of publications, respectively.

The vast majority of publications (94%, 76/81) reported the results of observational research studies employing retrospective chart reviews in facility settings. Only one publication provided quantitative data on intervention coverage or effectiveness among women or children, reporting the proportion of children and adolescents admitted to a trauma centre in Afghanistan who received physiotherapy.^40^

Explosive devices and remnants of war (eg, air or ground-launched explosive weapon or devices, improvised explosive devices, land mines) were the most commonly reported mechanism of injury, reported in 73 publications, 53 publications reported on injuries due to high-velocity/high-energy weapons and firearms. Burn injuries were reported in 13 publications, blunt trauma in 11 publications and crush injuries in 3 publications (table 1).

**Table 1:** Characteristics of included literature (n=81)

Study and population characteristics	n
Geographic region*	
East Asia and Pacific	4
Europe and Central Asia	10
Latin America and the Caribbean	1
Middle East and North Africa	27
Sub-Saharan Africa	9
South Asia	32
**Publication type**	
Non-research report	5
Observational study	76
Quasiexperimental study	0
Randomised controlled trial	0
**Target population type**	
All/general population	68
Children and adolescents	13
Displacement status of beneficiary population*	
Refugees	7
IDPs	4
Non-displaced	40
Returning refugees	2
Host	1
Unreported	30
Intervention delivery characteristics*	
Delivery platform*	
Existing health system	40
NGO/UN agencies	24
Military based	27
Research based	3
Intervention stage*	
Prehospital/triage/non-surgical management	56
Surgical management	76
Rehabilitation	19
Training/education	2
Mechanism of injury*	
Explosive devices and remnants of war	73
High-velocity/high-energy weapons/firearms	53
Burn injuries	13
Blunt trauma	11
Crush injuries	3

*Publications may be in more than one category.

IDP, internally displaced population; NGO, non-governmental organisation; UN, United Nations.

Reported interventions were grouped into four types or stages: (1) prehospital/triage/non-surgical management, which included wound management, burn care, pain management and fracture fixation; (2) surgical management, including amputations, abdominal surgery, cardiothoracic surgery, general surgery, neurosurgery, ophthalmic surgery and orthopaedic/reconstructive surgery; (3) rehabilitative services and management; and (4) training or education programmes.

**Figure 4:**
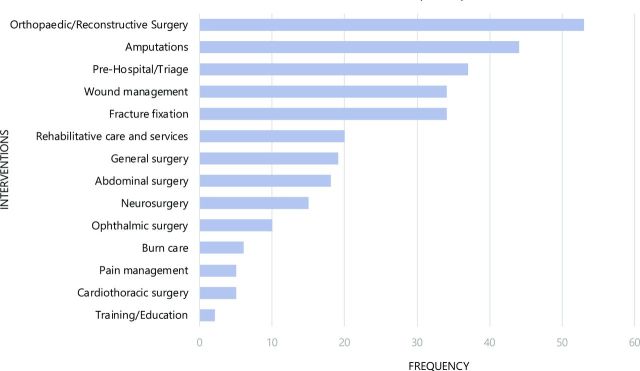
Frequency of delivered interventions reported in included publications (publications n=81, interventions n=302).

The most frequently reported interventions delivered were orthopaedic/reconstructive surgeries, described in 53 publications, while 44 publications discussed amputations and 34 publications discussed fracture fixations. The delivery of prehospital/triage care was reported in 37 publications, and wound care was reported in 34 publications (figure 4). The delivery of rehabilitative care and services was reported in only 19 publications, mostly referring to physiotherapy. Only five publications discussed the delivery of pain management interventions, and only six reported on burn care. One report discussed the delivery of a training intervention, which provided medical personnel with training in the management of emergency trauma cases and handling of mass casualties in Nepal, and another discussed a mine risk education programme offered to IDPs in Pakistan. This education programme was unique in our findings, as it was the only intervention reported to be delivered in community spaces, using print resources (including comic books for children), and involving community health workers, teachers and civic leaders as delivery personnel.

**Figure 5:**
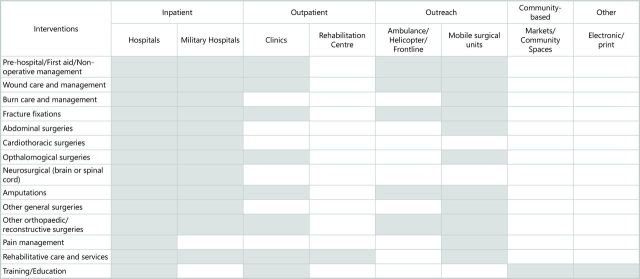
Reported site of delivery.

We classified sites of intervention delivery by their corresponding levels of care: inpatient, outpatient, outreach and community based (figure 5). Surgical procedures and non-operative management were predominately reported as being delivered in inpatient hospital settings (n=76). Sites at which outpatient care was provided included clinics where some prehospital/triage care and wound care interventions were delivered, as well as some fracture fixation, amputation and orthopaedic surgical interventions, and some ophthalmological surgeries. Other outpatient intervention delivery sites included specialised centres where rehabilitative care interventions were delivered. A range of trauma and rehabilitation interventions were reported as being delivered through outreach platforms such as mobile surgical units or in ambulances. Delivery site was unreported for at least five interventions described in the included literature.

**Figure 6:**
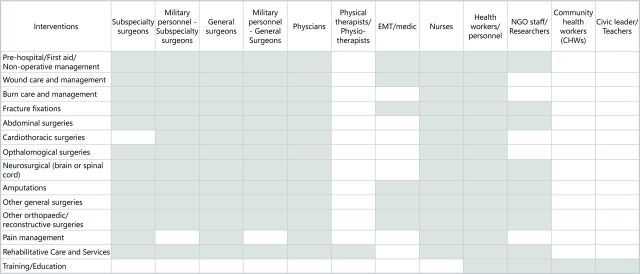
Reported delivery personnel. EMT, emergency medical technician; NGO, non-governmental organisation.

With respect to the personnel reported to be involved in the delivery of trauma and rehabilitation interventions, we found that highly specialised medical personnel were commonly reported across virtually all categories of interventions, including both civilian and military general and subspecialty surgeons, along with physicians, nurses and other trained health workers (figure 6). Paramedics or emergency medical technicians were also reported to be involved in the delivery of many intervention types, but less so for surgical interventions, while non-governmental organisations (NGO)/United Nations (UN) staff were reported as delivery personnel for some surgical interventions but not others, as well as for prehospital care and rehabilitative services. Physical therapists were also reported to be involved in the delivery of rehabilitative services.

Our review identified multiple elements that posed either as barriers or facilitators during the delivery of trauma and rehabilitation interventions (table 2). Commonly reported was the issue of evacuation to health facilities, with capacity to manage and stabilise trauma cases at the front line or site of injury and the distance and availability of transportation from the front line to the nearest health facility as key determinants of effective intervention delivery. A publication from Yemen reported the proximity of the hospital and the short evacuation time as enabling the effective treatment of severe vascular injuries similar to that achieved in a front-line hospital,^19^ while a publication from Colombia reported transportation delays and inefficient first response protocols as realities faced by Colombian trauma teams on a daily basis, increasing patient morbidity and mortality.^29^ The safety and security of health facilities themselves was another important determinant of service delivery. Two studies in Afghanistan noted that the bombing and closure of the Kunduz Trauma Center in 2015 resulted in a major loss for the local communities, leaving a wide gap in critical healthcare services and essential trauma care.^46 88^

**Table 2:** Barriers to and facilitators of intervention delivery

Issue	Barriers or facilitators
Evacuation and referral to health facilities	Capacity to manage and stabilise trauma cases at the front line/site of injury Distance to health facilities Availability of transportation Condition of roads and safety of routes Safety and security of health facilities
Expertise	Availability of skilled or specialised personnel for management of trauma cases Collaboration between domestic and international personnel; between military and humanitarian personnel
Supplies	Availability of appropriate medical supplies Availability of surgical equipment
Follow-up	Capacity for post-treatment follow-up of patients

Surgical procedures require trained personnel and injuries sustained due to warfare require specialised expertise for treatment, especially among paediatric patients. The availability of such skills and expertise, including through collaboration with other service providers, was another key determinant of intervention delivery reported in multiple publications. The availability of medical supplies and surgical equipment, including those appropriate for treating children, and the capacity to provide appropriate follow-up care were also commonly reported as important factors affecting intervention delivery.

## Discussion

This systematic review included 81 publications reporting on the delivery of trauma or rehabilitation interventions to conflict-affected populations including women, children and/or adolescents in 26 countries. Surgical interventions were the most commonly reported interventions, particularly orthopaedic surgeries, followed by prehospital care and wound management interventions. Relatively few publications reported on the delivery of rehabilitation interventions, and very few reported on the delivery of pain management interventions. Specialised medical personnel and skilled health workers, including both civilian and military, featured in the delivery of the whole range of trauma and rehabilitation interventions reported, with the exception of the few education interventions captured in this review. Most reported interventions were delivered in inpatient settings, but multiple examples of outpatient intervention delivery and delivery through mobile surgical units and other outreach modalities were also reported. The availability of emergency transport and access to health facilities was a major determinant of effective intervention delivery, as well as the availability of specialised medical personnel and supplies, and capacity for patient follow-up. Quantitative data on intervention coverage or effectiveness among women, children or adolescents particularly were generally unreported.

### Evidence gaps

Our findings suggest a number of key gaps in the literature with respect to the kinds of trauma and rehabilitation interventions and delivery modalities reported, as well as the kinds of conflict-affected populations featured in the literature.

Given the increase in both warfare and population displacement, prehospital management may now be more crucial than ever for the immediate management of trauma. Violent attacks are often unpredictable and designed to inflict maximum damage, causing widespread havoc in urban and rural areas and mass casualties that can quickly overwhelm local health facilities as well as transport and referral systems. The WHO-recommended trauma pathway^100^ proposes trauma stabilisation points (TSP) located near the active conflict zone for the initial assessment and triage of injuries. Ideally staffed by emergency medical teams, TSPs enable resuscitation and stabilisation of severely injured patients before referral to secondary or tertiary facilities and the immediate treatment and discharge of less severe patients. The need for evacuation teams, emergency medical teams and paramedics, and access to TSPs seems increasingly important to improve survival for victims,^101–104^ and yet the proportion of publications included in our review that reported on the delivery of prehospital care was considerably smaller than that reporting on the provision of surgical interventions and other procedures. It is unclear whether the fewer publications reporting on the delivery of prehospital care in our review reflect an actual intervention gap, in that such interventions are indeed delivered relatively infrequently, or rather that the *reporting* of such interventions is simply less frequent than the reporting of, for example, surgical interventions. Given the predominance in the literature of observational studies based on retrospective chart review particularly, the latter is certainly possible.

Recently, during the humanitarian health response to the siege of Mosul, Iraq, between October 2016 and July 2017, WHO identified the centrality of transport in its trauma management planning, noting that functioning referral pathways require viable ambulance networks with ‘care providers at the paramedic level or above during transport’ to provide en route care.^105^ In the current review, we found no publications reporting on innovative or otherwise successful approaches for improving the transport of the injured along the referral pathway.

Trauma and injuries due to armed conflict remain a major cause of morbidity and mortality worldwide,^4 106 107^ often with long-term complications. The overall estimated survival rate of improvised mine casualties in 2017 was reported to be 58%,^108^ and those who survive may have permanent disabilities and need comprehensive rehabilitative services such as physical therapy and the provision of orthotics or prostheses, as well as occupational therapy and mental health or psychosocial support to improve long-term quality of life. A significant proportion of publications included in this review reported injuries caused by land mines, explosive devices and ERWs, but only about one-quarter of included publications reported on the delivery of rehabilitative services. This is concerning, given these types of injuries can often require long-term follow-up and care to maintain functionality. Moreover, the majority of the captured literature on rehabilitation services in our review focused on physiotherapy, while complex injuries from ERWs often require physical therapy, occupational therapy and assisted devices. A recent systematic review on access to rehabilitation for people with disabilities in LMICs similarly found that coverage appeared to be low for medical rehabilitation, assistive devices, therapy and adherence.^10^ We also found no publications reporting on postoperative or post-treatment follow-up of injured patients. This too, however, may be a reflection of the discrepancy between those interventions reported in the literature and those conducted in the field.

Of the 81 publications included in our review, only 14 focused on children or adolescents as the target population for trauma or rehabilitation intervention delivery (although children and adolescents were generally included in the beneficiary populations reported on in most other included publications). Even from within this limited literature, however, some potentially useful insights emerge. The availability or lack of paediatric expertise and of medical supplies and surgical equipment appropriate for paediatric patients was reported as an important factor affecting intervention delivery, for example. An analysis of children with blast injuries in Afghanistan and Iraq concluded that documenting the particular surgical operative needs of child injuries promotes the development of plans to provide appropriate surgical care, helps guarantee the availability of age-appropriate surgical equipment and helps ensure predeployment education of the personnel who will provide humanitarian surgical care to paediatric populations.^36^ Elsewhere, organisations reported collaborating with other service providers to secure paediatric expertise, supplies and equipment. Such insights may point to potential priorities for operational research in this area.

### Limitations

In addition to the apparently limited documentation in the literature of trauma and rehabilitation intervention delivery for women and children in conflict settings, this review has other limitations. For logistical reasons, we restricted our review to reports published in English, and we conducted a comprehensive but not exhaustive search of the grey literature; both of these methodological decisions may have led to the exclusion of relevant publications providing different information on intervention delivery than what we have currently captured.

## Conclusion

Traumatic injuries require an array of medical and surgical interventions, and their effective treatment largely depends on prompt and timely management and referral, with appropriate rehabilitation services and post-treatment follow-up. The findings of our review provide some insight into where and how humanitarian health responders have been able to deliver this continuum of care to conflict-affected populations including women and children, and the challenges of doing so. However, the actual extent of trauma and rehabilitation intervention delivery in the field, the full range of delivery modalities being used and the effectiveness of those modalities are not comprehensively documented in the literature and remain largely unknown. Further work to evaluate intervention delivery in this domain is needed, particularly among children given their specialised needs, and in different population displacement contexts.

10.1136/bmjgh-2019-001980.supp1 Supplementary data
