# Supplementary material for: Delivering trauma and rehabilitation interventions to women and children in conflict settings: a systematic review
Source: BMJ Glob Health. 2020 Apr 23;5(Suppl 1):e001980. doi: 10.1136/bmjgh-2019-001980 (PMC7204922; doi:10.1136/bmjgh-2019-001980)
Supplement: Supplementary data [file bmjgh-2019-001980supp001.pdf]

APPENDIX A: Characteristics of included publications

Author	Country	Report Type	Intervention year	Age range	Intervention stage	Mechanism of Injury	Collapsed intervention	Delivery Platform	Site	Personnel
**Pediatric Population**										
Bertani 2015	Afghanistan	Observational study	2009	Children, Adolescents	Pre-Hospital/Triage/NOM, Surgical Management	ED, Guns/Weapons	Wound Management, Amputations, Fracture Fixation, Orthopedic/Reconstructive Surgery	Military	Hospitals	Subspecialty Surgeons
Can 2009	Turkey	Observational study	2001	Children, Adolescents	Pre-Hospital/Triage/NOM, Surgical Management	ED, Burns	Wound Management, Amputations, Ophthalmic Surgery	Existing health system	Hospitals	Not reported
Cleves 2016	Colombia	Observational study	2012	Children, Adolescents	Surgical Management	ED, Burns, Guns/Weapons, Blunt Trauma	General Surgery	Existing health system	Hospitals	Not reported
Coppola 2006	Iraq	Observational study	2004	Children, Adolescents	Pre-Hospital/Triage/NOM, Surgical Management	ED, Burns, Guns/Weapons	Wound Management, Fracture Fixation, Neurosurgery, Ophthalmic Surgery, Abdominal Surgery, Cardiothoracic Surgery, Orthopedic/Reconstructive Surgery	Military	Hospitals	General Surgeons, Subspecialty Surgeons, Physicians
Dua 2013	Iraq	Observational study	2006	Children, Adolescents	Pre-Hospital/Triage/NOM, Surgical Management	ED, Guns/Weapons	Pre-Hospital/Triage, Wound Management, Amputations, Orthopedic/Reconstructive Surgery	Military	Hospitals	Not reported
Edwards 2014	Afghanistan and Iraq	Observational study	2002	Children, Adolescents	Pre-Hospital/Triage/NOM, Surgical Management	ED	Wound Management, Amputations, Neurosurgery, Abdominal Surgery, Orthopedic/Reconstructive Surgery	Military	Hospitals	General Surgeons, Subspecialty Surgeons, Nurses

Jain RP, *et al. BMJ Global Health* 2020; 5:e001980. doi: 10.1136/bmjgh-2019-001980

Fares 2013	Lebanon	Observational study	2006	Children, Adolescents	Surgical Management	ED	Amputations, General Surgery	Existing health system	Hospitals	Not reported
Jandric 2001	Bosnia and Herzegovina	Observational study	1992	Children, Adolescents	Rehabilitation	ED	Rehabilitative care and services	Existing health system	Hospitals, Rehabilitation centers	Not reported
Mathieu 2015	Afghanistan	Observational study	2009	Children, Adolescents	Pre-Hospital/Triage/NOM, Surgical Management	ED, Guns/Weapons, Burns	Wound Management, Amputations, Fracture Fixation, Orthopedic/Reconstructive Surgery	Military	Hospitals	Subspecialty Surgeons
McKechnie 2014	Afghanistan	Observational study	2008	Children, Adolescents	Pre-Hospital/Triage/NOM, Surgical Management	ED, Guns/Weapons	Wound Management, Fracture Fixation, Amputations, Neurosurgery, Ophthalmic Surgery, Abdominal Surgery, Orthopedic/Reconstructive Surgery	Military	Hospitals	General Surgeons, Subspecialty Surgeons, Nurses
Villamaria 2014	Afghanistan and Iraq	Observational study	2002	Children, Adolescents	Surgical Management	ED, Guns/Weapons, Blunt Trauma	Amputations, Fracture Fixation, Abdominal Surgery, Cardiothoracic Surgery ,Orthopedic/Reconstructive Surgery	Military	Hospitals	Not reported
Wani 2011	India	Observational study	2001	Children, Adolescents	Pre-Hospital/Triage/NOM, Surgical Management	ED, Guns/Weapons	Pre-Hospital/Triage, Wound Management, Neurosurgery	Not reported	Hospitals	Subspecialty Surgeons
Wilson 2013	Afghanistan	Observational study	2010	Children, Adolescents	Pre-Hospital/Triage/NOM, Surgical Management	ED, Guns/Weapons, Burns	Pre-Hospital/Triage, Wound Management, Burn care, Abdominal Surgery Orthopedic/Reconstructive Surgery	Military	Hospitals	General Surgeons
Wu 2013	Kenya	Observational study	2005	Children, Adolescents	Pre-Hospital/Triage/NOM, Surgical Management	Not reported	Wound Management, General Surgery, Fracture Fixation, Orthopedic/Reconstructive Surgery	UN/NGO	Hospitals	Subspecialty Surgeons
**General Population**										

Jain RP, *et al. BMJ Global Health* 2020; 5:e001980. doi: 10.1136/bmjgh-2019-001980

Cutting 1992	Lebanon	Observational study	1985	Children, Adolescents, Women and Men	Pre-Hospital/Triage/NOM, Surgical Management	ED, Guns/Weapons	Pre-Hospital/Triage, Wound Management, Fracture Fixation, Amputations, Neurosurgery, Abdominal Surgery, Cardiothoracic Surgery, Orthopedic/Reconstructive Surgery, Pain Management, Rehabilitative care and services	UN/NGO	Hospitals	General Surgeons, Physicians, Nurses, Healthcare Workers, Physical therapists/Physiotherapists
Hemat 2017	Afghanistan	Observational study	2014	Children, Adolescents, Women and Men	Pre-Hospital/Triage/NOM, Surgical Management, Rehabilitation	ED, Guns/Weapons	Pre-Hospital/Triage, Fracture Fixation, Amputations, Neurosurgery, Abdominal Surgery, General Surgery, Orthopedic/Reconstructive Surgery, Rehabilitative care and services	UN/NGO	Hospitals, Clinics	Healthcare Workers, General Surgeons, Subspecialty Surgeons, Physicians, Healthcare Workers, NGO staff
Lacoux 2003	Sierra Leone	Observational study	2001	Children, Adolescents, Women and Men	Rehabilitation	Not reported	Pain Management	UN/NGO	Not reported	Not reported
Meade 2000	Sri Lanka	Observational study	1996	Children, Adolescents, Women and Men	Pre-Hospital/Triage/NOM, Surgical Management	ED, Guns/Weapons	Wound Management, Amputations, Ophthalmic Surgery, Abdominal Surgery, Orthopedic/Reconstructive Surgery	Existing health system UN/NGO, Research	Hospitals	General Surgeons, Nurses
Rashid 2010	India	Observational study	1988	Children, Adolescents, Women and Men	Pre-Hospital/Triage/NOM, Surgical Management	ED	Pre-Hospital/Triage, Wound Management, Orthopedic/Reconstructive Surgery	Existing health system	Hospitals	Not reported
Soroush 2010	Iran	Observational study	1988	Children, Adolescents and Women	Surgical Management	ED	Amputations	Existing health system	Ambulance/Helicopter/Battlefield, Hospitals	Not reported
Gousheh, J. 1995	Iran	Observational study	1979	Children, Adolescents, Women and Men	Pre-Hospital/Triage/NOM, Surgical Management	ED, Guns/Weapons	Wound Management, General Surgery, Orthopedic/Reconstructive	Existing health system, Research	Hospitals	Subspecialty Surgeon

Moreels, R., et al., 1994	Cambodia	Observational study	1990	Children, Adolescents, Women and Men	Pre-Hospital/Triage/NOM, Surgical Management	ED ,Guns/Weapons	Pre-Hospital/Triage, Wound Management, Abdominal Surgery, General Surgeries	Existing health system, UN/NGO	Hospitals	Physicians and General Surgeons
Strada, G., et al., 1993	Afghanistan	Observational study	1990	Children, Adolescents, Women and Men	Pre-Hospital/Triage/NOM, Surgical Management	ED, Guns/Weapons	Pre-Hospital/Triage, Amputation, Fracture Fixation, Abdominal Surgery, General Surgery	UN/NGO	Hospitals	General Surgeons, Doctors, Nurses,
Fakri 2012	Jordan	Observational study	2006	Children, Adolescents, Women and Men	Pre-Hospital/Triage/NOM, Surgical Management	ED, Guns/Weapons	Wound Management, Amputation, Fracture Fixation, Orthopedic/Reconstructive Surgery	UN/NGO	Hospitals	General Surgeons, Subspecialty Surgeons, Physicians,
UNICEF 2013	Pakistan	Non-Research	2011	Children, Adolescents, Women and Men	Training/Education	ED	Training/Education (Mine risk education)	UN/NGO	Markets/Community Spaces	Healthcare workers, NGO staff, CHW's, Teachers, Civic Leaders
Covey 2004	Sri Lanka	Observational study	1998	Adolescents, Women and Men	Pre-Hospital/Triage/NOM, Surgical Management, Rehabilitation	ED, Guns/Weapons	Pre-Hospital/Triage, Wound Management, Fracture Fixation, Orthopedic/Reconstructive Surgery, Rehabilitative care and services	Military, Existing health system	Hospitals	General Surgeons, Subspecialty Surgeons, Physicians, Healthcare Workers, Physical therapists/Physiotherapists
Khan 2002	Pakistan	Observational study	1990	Adolescents and Men	Surgical Management	ED	Amputations, Fracture Fixation, Orthopedic/Reconstructive Surgery	Not reported	Hospitals	Not reported
Hammer 1996	Somalia	Observational study	1993	Adolescents, Women and Men	Pre-Hospital/Triage/NOM, Surgical Management	Guns/Weapons	Wound Management, Fracture Fixation, Orthopedic/Reconstructive Surgery	Military, Research	Hospitals	General Surgeons
Al-Ganadi 2015	Yemen	Observational study	2011	General population	Pre-Hospital/Triage/NOM, Surgical Management	ED, Guns/Weapons	Pre-Hospital/Triage, Amputations, Orthopedic/Reconstructive Surgery	Existing health system	Hospitals	Not reported
Al-Nuaimi 2018	Turkey	Observational study	2012	General population	Rehabilitation	ED, Guns/Weapons	Rehabilitative care and services	Existing health system	Hospitals	Physicians, Healthcare Workers
Ascherio 1995	Mozambique	Observational study	1994	General population	Surgical Management, Rehabilitation	ED	Amputations, Rehabilitative care and services	Existing health system	Hospitals, Clinics	Not reported

Jain RP, *et al. BMJ Global Health* 2020; 5:e001980. doi: 10.1136/bmjgh-2019-001980

Atesalp 1999	Turkey	Observational study	1990	General population	Pre-Hospital/Triage/NOM, Surgical Management	ED	Pre-Hospital/Triage, Wound Management, Amputations, Orthopedic/Reconstructive Surgery	Existing health system	Hospitals	General Surgeons
Baldan 2014	Democratic Republic of Congo	Observational study	2007	General population	Pre-Hospital/Triage/NOM, Surgical Management, Rehabilitation	Not reported	Pre-Hospital/Triage, Wound Management, Fracture Fixation, Orthopedic/Reconstructive Surgery, Rehabilitative care and services	UN/NGO	Hospitals	General Surgeons, Nurses, Physical therapists/Physiotherapists
Bordes 2017	Afghanistan	Observational study	2010	General population	Surgical Management	ED, Guns/Weapons, Blunt Trauma	Abdominal Surgery, Cardiothoracic Surgery, Neurosurgery, Ophthalmic Surgery Orthopedic/Reconstructive Surgery	Military	Hospitals	General Surgeons, Physicans
Breeze 2011	Afghanistan	Observational study	2007	General population	Pre-Hospital/Triage/NOM, Surgical Management	ED	Wound Management, Amputations, Fracture Fixation, Neurosurgery, Ophthalmic Surgery, Abdominal Surgery, Orthopedic/Reconstructive Surgery	Military	Hospitals	General Surgeons, Subspecialty Surgeons, Physicians, Nurses
Bumbasirevic 2010	Serbia	Observational study	1991	General population	Pre-Hospital/Triage/NOM, Surgical Management, Rehabilitation	Not reported	Wound Management, Fracture Fixation, Orthopedic/Reconstructive Surgery, Rehabilitative care and services	Existing health system	Hospitals	Physicians
Davidovic 1997	Serbia	Observational study	1991	General population	Pre-Hospital/Triage/NOM, Surgical Management	ED, Guns/Weapons	Pre-Hospital/Triage, Amputations, Fracture Fixation, Orthopedic/Reconstructive Surgery	Existing health system	Hospitals	Not reported
Dua 2012	Afghanistan and Iraq	Observational study	2006	General population	Pre-Hospital/Triage/NOM, Surgical Management	ED, Guns/Weapons	Pre-Hospital/Triage, Wound Management Amputations, Orthopedic/Reconstructive Surgery	Military	Hospitals	General Surgeons

Jain RP, *et al. BMJ Global Health* 2020; 5:e001980. doi: 10.1136/bmjgh-2019-001980

Fares 2013	Lebanon	Observational study	2006	General population	Pre-Hospital/Triage/NOM, Surgical Management, Rehabilitation	ED	Pre-Hospital/Triage, Amputations, Neurosurgery, General Surgery, Pain Management,	Existing health system	Hospitals	Not reported
Gohy 2016	Afghanistan	Observational study	2015	General population	Pre-Hospital/Triage/NOM, Surgical Management, Rehabilitation	ED, Guns/Weapons, Blunt Trauma	Pre-Hospital/Triage, Fracture Fixation ,General Surgery, Rehabilitative care and services	UN/NGO	Hospitals, Clinics	Physical therapist/ Physiotherapists
Gosselin 1993	Pakistan	Observational study	1989	General population	Pre-Hospital/Triage/NOM, Surgical Management	ED, Guns/Weapons, Crush Injuries	Pre-Hospital/Triage, Amputations, Orthopedic/Reconstructive Surgery	UN/NGO	Hospitals	General Surgeons, Subspecialty Surgeons
Gousheh 2008	Iran	Observational study	1980	General population	Surgical Management	ED, Guns/Weapons	Orthopedic/Reconstructive Surgery	Existing health system	Hospitals	Physicians
Guerrier 2015	Jordan	Observational study	2006	General population	Pre-Hospital/Triage/NOM, Surgical Management	ED, Guns/Weapons	Pre-Hospital/Triage, Wound Management, Fracture Fixation, Orthopedic/Reconstructive Surgery	Existing health system, UN/NGO	Hospitals	General Surgeons, Subspecialty Surgeons
Hanevik 2000	Eritrea	Observational study	1991	General population	Surgical Management	ED	Amputations, Orthopedic/Reconstructive Surgery	Existing health system	Hospitals	Not reported
Henning 2011	Afghanistan	Observational study	2008	General population	Pre-Hospital/Triage/NOM, Surgical Management	ED, Guns/Weapons, Burns, Crush Injuries	Pre-Hospital/Triage, General Surgery, Orthopedic/Reconstructive Surgery	Military	Ambulance/Helicopter/Battlefield, Hospital	Physicians, Nurses, EMT/Medic
Heszlein-Lossius 2018	Palestine	Observational study	2006	General population	Rehabilitation	ED	Rehabilitative care and services	UN/NGO	Rehabilitation centers	Not reported
Hettiaratchy 1996	Afghanistan	Observational study	1994	General population	Surgical Management, Rehabilitation	Not reported	Amputations, Rehabilitative care and services	UN/NGO	Clinics	Not reported
Hornez 2015	Jordan	Observational study	2013	General population	Pre-Hospital/Triage/NOM, Surgical Management	ED, Guns/Weapons, Burns	Pre-Hospital/Triage, Fracture Fixation, Amputations, Abdominal Surgery, General Surgery Orthopedic/Reconstructive Surgery, Pain Management	Military, Existing health system, UN/NGO	Hospitals, Mobile Surgical Units	General Surgeons, Subspecialty Surgeons, Physicians, Nurses,

Jain RP, *et al. BMJ Global Health* 2020; 5:e001980. doi: 10.1136/bmjgh-2019-001980

Host 2012	Cambodia	Non-Research	2003	General population	Surgical Management	ED, Burns	General Surgery	UN/NGO	Hospitals	Not reported
Hougen 2000	Thailand	Observational study	1997	General population	Pre-Hospital/Triage/NOM, Surgical Management, Rehabilitation	ED	Pre-hospital/Triage, Amputations, General Surgery, Rehabilitative Care and Services	Not reported	Hospitals	Not reported
Hussain 2001	Pakistan	Observational study	1995	General population	Surgical Management	ED, Guns/Weapons	Amputations, Fracture Fixation, Orthopedic/Reconstructive Surgery	Existing health system	Hospitals	Not reported
Irmay 2000	Sierra Leone	Observational study	1998	General population	Pre-Hospital/Triage/NOM, Surgical Management, Rehabilitation	ED, Guns/Weapons, Burns	Pre-hospital/Triage, Wound Management, Amputations, General Surgery, Orthopedic/Reconstructive Surgery, Rehabilitative care and services	Existing health system, UN/NGO	Hospitals	General Surgeons, Subspecialty Surgeons
Jacobs 2012	Afghanistan	Observational study	2008	General population	Pre-Hospital/Triage/NOM, Surgical Management	ED	Pre-Hospital/Triage, Burn care, Wound Management, Amputations, Fracture Fixation, Neurosurgery, Ophthalmic Surgery, Abdominal Surgery, Cardiothoracic Surgery, General Surgery, Orthopedic/Reconstructive Surgery,	Military	Hospitals	Physicians
Jaouni 1997	Palestine	Observational study	1987	General population	Pre-Hospital/Triage/NOM, Surgical Management	ED, Guns/Weapons	Pre-Hospital/Triage, Ophthalmic Surgery	Existing health system	Hospitals, Clinics	Physicians
Joubert 2016	Afghanistan	Observational study	2010	General population	Surgical Management	ED, Guns/Weapons	Fracture Fixation, Neurosurgery, Ophthalmic Surgery, General Surgery, Orthopedic/Reconstructive Surgery	Existing health system	Hospitals	Subspecialty Surgeons, Physicians
Khattak 2015	Pakistan	Observational study	2012	General population	Surgical Management	ED, Guns/Weapons, Burns, Blunt Trauma	General Surgery	Existing health system	Hospitals	General Surgeons, Subspecialty Surgeons
Korver 1993	Pakistan	Observational study	1990	General population	Surgical Management	Guns/Weapons, Burns	Amputations	UN/NGO	Hospitals	NGO staff

Kummoona 2010	Iraq	Observational study	1990	General population	Pre-Hospital/Triage/NOM, Surgical Management	Guns/Weapons	Pre-Hospital/Triage, Fracture Fixation, Neurosurgery, Ophthalmic Surgery, General Surgery, Orthopedic/Reconstructive Surgery	Existing health system	Hospitals	General Surgeons, Subspecialty Surgeons
Lacoux 2002	Sierra Leone	Observational study	2000	General population	Surgical Management	Guns/Weapons	Amputations	Existing health system	Hospitals	Not reported
Madi 2015	Algeria	Non-Research	2007	General population	Rehabilitation	Not reported	Rehabilitative care and services	Existing health system, UN/NGO	Rehabilitation centers	Not reported
Malgras 2015	Central African Republic	Observational study	2013	General population	Pre-Hospital/Triage/NOM, Surgical Management	ED, Guns/Weapons	Pre-Hospital/Triage, Orthopedic/Reconstructive Surgery	Military, UN/NGO	Ambulance/Helicopter/Battlefield, Mobile Surgical Units	General Surgeons, Subspecialty Surgeons, Nurses, NGO staff, EMT/Medic
Maricevic 1997	Bosnia and Herzegovina	Observational study	1991	General population	Pre-Hospital/Triage/NOM, Surgical Management, Rehabilitation	ED, Guns/Weapons	Pre-Hospital/Triage, Wound Management, Amputations, Fracture Fixation, General Surgery, Orthopedic/Reconstructive Surgery, Rehabilitative care and services	Existing health system	Ambulance/Helicopter/Battlefield, Hospitals	General Surgeons, Subspecialty Surgeons, Physicians, Nurses, Healthcare Workers, EMT/Medic Physical therapists/Physiotherapists,
Mathieu 2014	Afghanistan	Observational study	2009	General population	Pre-Hospital/Triage/NOM, Surgical Management	ED, Guns/Weapons, Burns	Wound Management, Amputations, Fracture Fixation, Orthopedic/Reconstructive Surgery	Military	Hospitals	General Surgeons, Subspecialty Surgeons, EMT/Medic
Mitkovic 2013	Serbia	Observational study	NR	General population	Pre-Hospital/Triage/NOM, Surgical Management, Rehabilitation	ED	Pre-Hospital/Triage ,Wound Management, Amputations, Fracture Fixation, Orthopedic/Reconstructive Surgery, Rehabilitative care and services	Existing health system	Clinics	General Surgeons, Subspecialty Surgeons

Nikolic 2000	Serbia	Observational study	1991	General population	Pre-Hospital/Triage/NOM, Surgical Management	ED, Guns/Weapons	Pre-Hospital/Triage, Wound Management, Amputations, Fracture Fixation, Orthopedic/Reconstructive Surgery	Not reported	Hospitals	Not reported
Peck 2007	Iraq	Observational study	2004	General population	Pre-Hospital/Triage/NOM, Surgical Management	ED, Guns/Weapons	Wound Management, Amputations, Orthopedic/Reconstructive Surgery	Military	Hospitals	General Surgeons
Ramasamy 2010	Afghanistan	Observational study	2006	General population	Pre-Hospital/Triage/NOM, Surgical Management	ED, Burns	Burn care, Amputations, Fracture Fixation, Neurosurgery, Abdominal Surgery, Orthopedic/Reconstructive Surgery	Military	Hospitals	General Surgeons, Subspecialty Surgeons
Ravindra 2016	Afghanistan	Observational study	2007	General population	Surgical Management	ED, Guns/Weapons, Blunt Trauma	Fracture Fixation	Military	Hospitals	Subspecialty Surgeons
Sfeir 1995	Lebanon	Observational study	1975	General population	Pre-Hospital/Triage/NOM, Surgical Management	ED, Guns/Weapons, Blunt Trauma	Pre-Hospital/Triage, Amputations, Orthopedic/Reconstructive Surgery	Existing health system	Hospitals	Not reported
Sfeir 1992	Lebanon	Observational study	1975	General population	Pre-Hospital/Triage/NOM, Surgical Management	ED, Guns/Weapons, Blunt Trauma	Pre-Hospital/Triage, Amputations, Fracture Fixation, Other Orthopedic/Reconstructive Surgery	Existing health system	Hospitals	Subspecialty Surgeons
Shabila 2010	Iraq	Observational study	1998	General population	Surgical Management	ED	Amputations	Existing health system	Hospitals	Not reported
Taha 1991	Lebanon	Observational study	1981	General population	Pre-Hospital/Triage/NOM, Surgical Management	ED, Guns/Weapons	Pre-Hospital/Triage, Wound Management, Neurosurgery, Orthopedic/Reconstructive Surgery	Existing health system	Ambulance/Helicopter/Battlefield, Hospitals	Not reported

Jain RP, *et al. BMJ Global Health* 2020; 5:e001980. doi: 10.1136/bmjgh-2019-001980

Trelles 2016	Afghanistan	Observational study	2011	General population	Pre-Hospital/Triage/NOM, Surgical Management	ED, Guns/Weapons, Blunt Trauma	Pre-Hospital/Triage, Wound Management, Burn care, Amputations, Fracture Fixation, Abdominal Surgery, General Surgery ,Other Orthopedic/Reconstructive Surgery	UN/NGO	Hospitals	General Surgeons, Subspecialty Surgeons, Physicians, Nurses, Healthcare Workers
Uruc 2014	Turkey	Observational study	2011	General population	Pre-Hospital/Triage/NOM, Surgical Management	ED, Guns/Weapons, Crush Injuries, Blunt Trauma	Pre-Hospital/Triage, Wound Management, Burn care, Amputations, Fracture Fixation, Other Orthopedic/Reconstructive Surgery	Existing health system	Hospitals	Not reported
Woll 2013	Afghanistan	Observational study	2011	General population	Pre-Hospital/Triage/NOM, Surgical Management	ED, Guns/Weapons	Pre-Hospital/Triage, Wound Management, Burn care, Fracture Fixation, Ophthalmic Surgery, Abdominal Surgery, General Surgery, Orthopedic/Reconstructive Surgery,	Military	Hospitals, Mobile surgical units	General Surgeons, Military subspecialty Surgeons, Physicians, Healthcare Workers
Younis 2011	Palestine	Observational study	2006	General population	Surgical Management, Rehabilitation	ED, Guns/Weapons, Blunt Trauma	Neurosurgery, Rehabilitative care and services	Existing health system	Hospitals	Not reported
Leininger, B.E., et al. 2006	Iraq	Observational study	2004	General population	Pre-Hospital/Triage/NOM, Surgical Management	ED, Guns/Weapons	Wound Management, Orthopedic/Reconstructive Surgery	Military	Hospitals	General Surgeons, Physicians, Nurses, Healthcare workers
Roostar, L., 1995	Afghanistan	Observational study	NR	General population	Pre-Hospital/Triage/NOM, Surgical Management	ED, Guns/Weapons	Pre-Hospital/Triage, Amputations, Orthopedic/Reconstructive Surgery	Existing health system	Hospitals	Not reported
Zangana, A.M., 2007	Iraq	Observational study	2003	General population	Pre-Hospital/Triage/NOM, Surgical Management, Rehabilitation	ED, Guns/Weapons	Pre-Hospital/Triage, Wound Management, Abdominal Surgery ,General Surgery, Pain Management	Existing health system	Hospitals	General Surgeons, Physicians, Nurses, Healthcare workers,

ICRC 2013	Nepal	Non-Research	1998	General population	Rehabilitation, Training/Education	ED, Guns/Weapons	Rehabilitative care and services, Training/Education (Training Medical personnel in the management of Emergency trauma cases and handling of mass casualties).	Military, Existing health system, UN/NGO	Hospitals, Clinics	NGO staff
ICRC 2016	Philippines	Non-Research	2008	General population	Rehabilitation	Not reported	Rehabilitative care and services	UN/NGO	Clinics	Healthcare workers

Appendix B. MEDLINE search strategy

Conflict related terms

1. Medline

1. disasters/ or emergencies/ or mass casualty incidents/
2. disaster victims/
3. ((disaster or disasters or catastrophe or catastrophes) adj5 (environ* or human or manmade or "man made" or nature or natural or weather)).tw,kf.
4. ("mass casualty" or "mass casualties" or "mass fatalities" or "mass fatality").tw,kf.
5. ((crisis or crises) adj5 (environ* or human or manmade or "man made" or nature or natural or weather)).tw,kf.
6. "warfare and armed conflicts"/ or armed conflicts/ or warfare/ or biological warfare/ or bioterrorism/ or chemical warfare/ or chemical terrorism/ or nuclear warfare/ or psychological warfare/ or war crimes/ or ethnic cleansing/ or genocide/ or holocaust/ or war exposure/ or war-related injuries/
7. afghan campaign 2001-/ or gulf war/ or iraq war, 2003-2011/
8. ("afghan campaign" or "armed conflict" or "armed conflicts" or "gulf war" or "iraq war" or "war time" or "wartime").tw,kf.
9. ((armed or zone or political or civil) adj3 (conflict or conflicts or attack or attacks or war or wars or "no fly")).tw,kf.
10. ("war related injuries" or "war related traumas" or "war related injury" or "war related trauma").tw,kf.
11. ("militant group" or "militant groups" or "militant organization" or "militant organizations" or "militant organisation" or "militant organisations").tw,kf.
12. ("biological terrorism" or bioterrorism or biowarfare or "chemical terrorism" or "ethnic cleansing" or "ethnic cleansings" or "gas poisoning" or genocide or holocaust or holocausts or "nuclear terrorism" or "war exposure" or "war exposures").tw,kf.
13. Disaster Medicine/
14. disease outbreaks/
15. Emergency Medical Services/
16. ((emergency or emergencies) adj5 (environ* or human or manmade or "man made" or nature or natural or weather)).tw,kf.
17. Starvation/
18. (famine or famines or starvation or starvations).tw,kf.
19. cyclonic storms/ or droughts/ or floods/ or tornadoes/ or tidal waves/
20. avalanches/ or earthquakes/ or landslides/ or tidal waves/ or tsunamis/ or volcanic eruptions/
21. (avalanche or avalanches or cyclone or cyclones or drought or droughts or earthquake or earthquakes or flood or flooded or flooding or floods or hurricane or hurricanes or landslide or landslides or "land slide" or "land slides" or mudslide or mudslides or "mud slide" or "mud slides" or storm or storms or tornado or tornadoes or tsunami or tsunamis or typhoon or typhoons or "volcanic ash" or "volcanic eruption" or "volcanic eruptions" or "volcanic gases").tw,kf.
22. refugees/
23. (evacuee or evacuees or refugee or refugees or squatter or squatters or transients).tw,kf.

24. relief work/ or rescue work/
25. ((rescue or relief or aid) adj (plan or plans or activity or activities or agency or agencies)).tw,kf.
26. ("aid plan" or "aid work" or "relief plan" or "relief work" or "rescue plan" or "rescue work").tw,kf.
27. ((staff or staffs or worker or workers) adj3 (relief or aid)).tw,kf.
28. (humanitarian assistance or humanitarian assistances or relief work or relief works).tw,kf.
29. (humanitarian adj2 (aid or response or relief or crisis or crises or emergency or emergencies or disaster or disasters)).tw,kf.
30. Altruism/
31. (humanitarianism or altruism).tw,kf.
32. ("displaced children" or "displaced families" or "displaced family" or "displaced individuals" or "displaced internally" or "displaced men" or "displaced people" or "displaced peoples" or "displaced person" or "displaced persons" or "displaced population" or "displaced populations" or "displaced women" or "forced displacement" or "forced displacements" or "internal displaced" or "internal displacement" or "internally displaced" or "population displaced" or "population displacement").tw,kf.
33. (((camp or camps) and displac*) or "protected village*").tw,kf.
34. (victim or victims).tw,kf.
35. rubble.tw,kf.
36. or/1-35

Population of interest

1. MEDLINE

37. adolescent/ or young adult/

38. (adolescence or adolescent or adolescents or teen* or youth or youths or "young adult" or "young adults").tw,kf.

39. Pregnant Women/

40. exp pregnancy/

41. (expectant or expectancy or gravid* or pregnant or pregnancies or pregnancy).tw,kf.

42. ("mother to be" or "mothers to be").tw,kf.

43. (prenatal or "pre natal").mp.

44. (perinatal or "peri natal").mp.

45. ((trimester or trimesters) adj3 (first or second or mid or third or final or "1st" or "2nd" or "3rd")).tw,kf.

46. (midtrimester or midtrimesters or "early placental phase" or "early placental phases").tw,kf.

47. exp Delivery, Obstetric/

48. ((labor or labour) adj5 (birth* or breech or childbirth or childbirths or complicat* or difficult or early or easy or induce* or induction or late or obstetric* or onset or pregnan* or present*)).tw,kf.

49. parturients.tw,kf.

50. (birth or births or childbirth or childbirths or parturition or parturitions).tw,kf.

51. ("abdominal deliveries" or "abdominal delivery" or "c-section" or "c-sections" or caesarean or caesareans or cesarean or cesareans or "postcesarean section" or "postcaesarean section").tw,kf.

52. exp Abortion, Induced/

53. (abortion or abortions or embryotomies or embryotomy or "postconception fertility control").tw,kf.

54. ((pregnancy or pregnancies) adj3 terminat*).tw,kf.

55. "sexually active".tw,kf.

56. child/ or child, preschool/ or infant/ or infant, newborn/ or infant, low birth weight/ or infant, small for gestational age/ or infant, very low birth weight/ or infant, extremely low birth weight/ or infant, postmature/ or infant, premature/ or infant, extremely premature/

57. (infan* or newborn* or "new born*" or neonat* or baby* or babies or toddler* or boy or boys or boyfriend or boyhood or girl* or kid or kids or child* or pediatric* or paediatric* or peadiatric* or prematur* or preterm*).mp. or school*.tw.

58. refugees/

59. (refugee or refugees).tw,kf.

60. or/37-59

61. 36 and 60

Domain specific terms – Injury & Rehabilitation

1. MEDLINE

62. Amputees/

63. amputation/ or disarticulation/

64. Amputation Stumps/

65. Amputation, Traumatic/

66. (amputat* or amputee or amputees or disarticulat* or exarticulat* or reamput*).tw,kf.

67. Visually Impaired Persons/

68. Blindness/

69. (amaurosis or blindness or "impaired sight" or "impaired vision" or "sight impairment" or "vision defect" or "vision defects" or "visual* impair*" or "visual loss" or "vision loss").tw,kf.

70. brain injuries/ or exp brain hemorrhage, traumatic/ or exp brain injuries, diffuse/ or exp brain injuries, traumatic/ or exp brain injury, chronic/ or epilepsy, post-traumatic/ or pneumocephalus/

71. ("brain commotion" or "brain concussion" or "brain concussions" or "brain contusion" or "brain contusions" or "brain damage" or "brain hemorrhage" or "brain hemorrhages" or "brain lesion" or "brain stem hematoma" or "brain stem hemorrhage" or "brain stem injury" or "brain stem lesion" or "brain stem trauma" or "brain system trauma " or "brain trauma" or "brain traumas" or "brainstem hematoma" or "brainstem hematomas" or "brainstem injury" or "bulbar hemorrhage" or "cerebellar contusion" or "cerebellar contusions" or "cerebellar damage" or "cerebellar hemorrhage" or "cerebellar hemorrhages" or "cerebellar lesion" or "cerebellum damage" or "cerebellum injury" or "cerebellum lesion" or "cerebellum trauma" or "cerebral concussion" or "cerebral concussions" or "cerebral contusion" or "cerebral contusions" or "cerebral damage" or "cerebral hematoma" or "cerebral hematomas" or "cerebral hemorrhage" or "cerebral hemorrhages" or "cerebral injuries" or "cerebral injury" or "cerebral intraparenchymal hematoma*" or "cerebral intraparenchymal hemorrhage*" or "cerebral lesion" or "cerebral parenchymal hemorrhage*" or "concussive convulsion" or "concussive convulsions" or "contusio cerebri" or "cortical contusion" or "cortical contusions" or "cranial airocele" or "cranial airoceles" or "cranial pneumocyst" or "cranial pneumocysts" or "damaged brain" or "dementia pugulistica" or "diffuse axonal injuries" or "diffuse axonal injury" or "early post traumatic seizure" or "early post traumatic seizures" or "forebrain lesion" or "head contusion" or "hemisphere lesion" or "impact seizure" or "impact seizures" or "intermediate concussion" or "intermediate concussions" or "intracerebral hemorrhage" or "intracerebral hemorrhages" or "intracranial lesion" or "late post traumatic seizure" or "late post traumatic seizures" or "mild concussion" or "mild concussions" or "pneumocephalus" or "post concussion symptom*" or "post concussion syndrome*" or "post concussive chronic encephalopathies" or "post concussive chronic encephalopathy" or "post concussive encephalopath*" or "post concussive encephalopathies" or "post concussive encephalopathy" or "post concussive syndrome" or "post traumatic encephalopath*" or "post traumatic encephalopathies" or "post traumatic encephalopathy" or "post traumatic epilepsies" or "post traumatic epilepsy" or "post traumatic seizure disorder" or "post traumatic seizure disorder*" or "post traumatic seizure disorders" or "postconcussion symptom*" or "postconcussion syndrome*" or "postconcussive syndrome" or "severe concussion" or "severe concussions" or "shaken baby" or "traumatic brain lesion" or "traumatic cerebral lesion" or "traumatic encephalopathies" or "traumatic encephalopathy" or "traumatic epilepsies" or "traumatic epilepsy").tw,kf.

72. Cerebral Palsy/

73. ("brain palsy" or "brain paralysis" or "central palsy" or "central paralysis" or "cerebral palsies" or "cerebral palsy" or "cerebral paralysis" or "cerebral paresis" or "encephalopathia infantilis" or "little disease" or "little's disease" or "spastic diplegia" or "spastic diplegias").tw,kf.

74. Developmental Disabilities/

75. ("abnormal development" or "child development deviation" or "child development deviations" or "child development disorder" or "child development disorders" or "development delay" or "development disorder" or "developmental delay" or "developmental disabilities" or "developmental disability").tw,kf.

76. Clubfoot/

77. (clubfeet or clubfoot or equinovarus or "pie torcido" or "pie torcidos").tw,kf.

78. communication disorders/ or language disorders/ or language development disorders/ or speech disorders/ or aphasia/ or articulation disorders/ or dysarthria/ or echolalia/ or mutism/ or stuttering/

79. (alalia or alogia or alogias or anepia or anepias or aphasia or aphasias or aprosodia or aprosodias or "aprosodic speech" or "articulation disorder" or "articulation disorders" or cluttering or clutterings or "communication disabilities" or "communication disability" or "communication disease" or "communication disorder" or "communication disorders" or "communication problem" or "communicative disorder" or "communicative disorders" or "communicative dysfunction" or "communicative dysfunctions" or "delayed language" or "delayed speech" or "disarticulation disorder" or "disarticulation disorders" or dysarthoses or dysarthosis or dysarthria or dysarthrias or dysglossia or dysglossias or dyslalia or dyslalias or dysphasia or "echo reaction" or "echo speech" or "echolalia" or "echophrasia" or "language deficiency" or "language delay" or "language delays" or "language development disorder" or "language development disorders" or "language disability" or "language disorder" or "language disorders" or "language impairment" or "late talker" or logagnosia or logagnosias or logamnesia or logamnesias or logasthenia or logasthenias or misarticulation or mutism or mutisms or "phonological impairment" or "phonological impairments" or "phonology impairment" or "phonology impairments" or rhinolalia or rhinolalias or "semantic pragmatic disorder" or "semantic pragmatic disorders" or "speech articulation disorder" or "speech articulation disorders" or "speech delay" or "speech delays" or "speech disorders" or "speech disorder" or stammering or stuttering or "unintelligible articulation" or "unintelligible articulations" or "verbal fluency disorder" or "verbal fluency disorders").tw,kf.

80. (crutch or crutches).tw,kf.

81. Persons With Hearing Impairments/

82. Deafness/

83. Hearing Loss/

84. ("auditorily impaired" or "auditory defect " or "auditory impaired" or deaf or deafness or "hard of hearing" or "hearing damage" or "hearing defect" or "hearing difficult* " or "hearing

impaired" or "hearing impairment" or "hearing impairments" or "hearing loss" or hypacusia or hypacusis or hypoacousia or hypoacuses or hypoacusis or "impaired hearing").tw,kf.

85. (disablement or disability or disabilities or handicap*).tw,kf.

86. Disabled Persons/

87. ("disabled patient" or "disabled person" or "disabled persons" or "physically challenged" or "physically disabled").tw,kf.

88. Disabled Children/

89. ("disabled child" or "disabled children").tw,kf.

90. Mentally Disabled Persons/

91. ("cognitively challenged person" or "cognitively deficient person" or "cognitively disabled person" or "cognitively impaired person" or "cognitively retarded person" or "intellectually challenged person" or "intellectually deficient person" or "intellectually disabled person" or "intellectually impaired person" or "intellectually retarded person" or "mental retards" or "mentally challenged person" or "mentally deficient person" or "mentally impaired person" or "mentally retarded").tw,kf.

92. Hemiplegia/

93. (hemiparalysis or hemiplagia or hemiplagias or hemiplegic or hemiplegy or monoplegia or monoplegias or "hemi plagia" or "hemi plagias" or "mono plegia" or "mono plegias" or "hemi paralysis" or "hemi plegic" or "hemi plegy").tw,kf.

94. Intellectual Disability/

95. ("cognitive retard" or "cognitive retardation" or idiocy or "intellectual development disorder" or "intellectual development disorders" or "intellectual dysfunction" or "intellectual retardation" or "mental deficiencies" or "mental deficiency" or "mental deficit" or "mental incapacity" or "mental retard" or "mental retardation" or "mentally retarded").tw,kf.

96. Learning Disorders/

97. ("developmental academic disorder" or "developmental academic disorders" or "developmental disorders of scholastic skills" or "impaired learning" or "learning deficit" or "learning difficulty" or "learning disabilities" or "learning disability" or "learning disorder" or "learning disorders" or "learning disturbance" or "learning disturbances" or "learning impairment" or "learning problem" or "scholastic skills development disorders").tw,kf.

98. Muscular Dystrophies/

99. ("muscle dystrophia" or "muscle dystrophy" or "muscular dystrophia" or "muscular dystrophies" or "muscular dystrophy" or myodystrophia or myodystrophica or myodystrophicas or myodystrophies or myodystrophy).tw,kf.

100. musculoskeletal abnormalities/

101. ("anomalous muscle" or "bone anomalies" or "bone anomaly" or "bone deformities" or "bone deformity" or "joint malformation*" or "muscle malformation*" or "muscle anomalies" or "muscle anomaly" or "musculo skeletal abnormalities" or "musculo skeletal abnormality" or "musculoskeletal abnormalities" or "musculoskeletal abnormality" or "ossification disturbance" or "osteoarticular anomaly").tw,kf.

102. Orthopedics/

103. (orthopaedia or orthopaedic or orthopaedics or orthopedic or orthopedics or orthopedy).tw,kf.

104. orthotic devices/ or athletic tape/ or braces/ or foot orthoses/

105. ("athletic tape" or brace or braces or "foot arch support" or "foot arch supports" or "kinesio tape" or "kinesio tapes" or kinesiotape or "orthopaedic footwear" or "orthopaedic shoe" or "orthopaedic shoes" or "orthopedic footwear" or "orthopedic shoe" or "orthopedic shoes" or orthos or orthoses or orthosis or "orthotic device" or "orthotic devices" or "orthotic insole" or "orthotic insoles" or "orthotic shoe insert" or "orthotic shoe inserts" or parapodium or parapodiums).tw,kf.

106. Paraplegia/

107. (paraplegia or paraplegias or paraplegic).tw,kf.

108. ("physical deficiency" or "physical disability assessment" or "physical disability evaluation" or "physical handicap" or "physical incapacity" or "physically disabled" or "physically handicapped").tw,kf.

109. physical therapy modalities/ or exp animal assisted therapy/ or drainage, postural/ or exp electric stimulation therapy/ or exp exercise movement techniques/ or exp exercise therapy/ or extracorporeal shockwave therapy/ or exp hydrotherapy/ or exp musculoskeletal manipulations/ or myofunctional therapy/

110. ("active stretching" or "acupressure" or "alexander technique" or "analgesic cutaneous electrostimulation" or "animal assisted therapies" or "animal assisted therapy" or "animal facilitated therapies" or "animal facilitated therapy" or "applied kinesiology" or "arm exercise" or "ballistic stretching" or "bodywork" or "bodyworks" or "breathing exercises" or "breathing therapy" or "cervical manipulation" or "chest wall oscillation" or "ch'i kung" or "chi, tai" or "chih ya" or "closed kinetic chain exercise" or "cpm therapies" or "cpm therapy" or "dance therapies" or "dance therapy" or "douching" or "douchings" or "dynamic exercise" or "dynamic stretching" or "electric stimulation therapy" or "electrical stimulation therapy" or "electro therapy" or "electroacupuncture" or "electroanalgesia" or "electroanalgesias" or "electrotherapy" or "equine assisted psychotherapies" or "equine assisted psychotherapy" or "equine assisted therapies" or "equine assisted therapy" or "equine assisted therapy" or "exercise movement technic" or "exercise movement technics" or "exercise movement technique" or "exercise movement techniques" or "exercise recovery" or "exercise therapies" or "exercise therapy" or "exercise treatment" or "exercise treatments" or "extracorporeal high intensity focused ultrasound therapy" or "extracorporeal shockwave therapy" or "feldenkrais method").tw,kf.

111. ("hellerwork" or "hifu therapies" or "hifu therapy" or "high intensity focused ultrasound ablation" or "high intensity focused ultrasound therapies" or "high intensity focused ultrasound therapy" or "hippotherapies" or "hippotherapy" or "horseback riding therapies" or "horseback riding therapy" or "hydrotherapies" or "hydrotherapy" or "interferential current electrotherapy" or "isokinetic exercise" or "isometric endurance" or "isometric exercise" or "isometric stretching" or "isometric training" or "ji quan, tai" or "joint mobilisation" or "joint mobilization" or "kegel exercise" or "kegel exercises" or "kinesiotherapy" or "kinesitherapy" or "kneipp therapy" or "kneipp treatment" or "lavage" or "lavages" or "leg exercise" or "lumbar manipulation" or "manipulation therapies" or "manipulation therapy" or "manipulative therapies" or "manipulative therapy" or "manual lymph drainage" or "manual lymphatic drainage" or "manual therapies" or "manual therapy" or "massage" or "massages" or "motion therapy" or "movement therapy" or "movement therapies" or "muscle strengthening" or "muscle training" or "musculoskeletal manipulation" or "musculoskeletal manipulations" or "myofunctional therapies" or "myofunctional therapy" or "neuromuscular facilitation" or "open kinetic chain exercise" or "oral myotherapies" or "oral myotherapy" or "orofacial myologies" or "orofacial myology" or "orofacial myotherapies" or "orofacial myotherapy" or "orthopaedic manipulation*" or "orthopedic manipulation*" or "osteopathic manipulation*" or "osteopathic manipulative treatment" or "osteopathic manipulative treatments").tw,kf.

112. ("passive stretching" or "pelvic floor exercise" or "pelvic floor exercises" or "pelvic floor muscle exercise" or "pelvic floor muscle exercises" or "pelvic floor training" or "pelvic muscle exercise" or "pelvic muscle exercises" or "percutaneous electric nerve stimulation*" or "percutaneous electrical nerve stimulation*" or "percutaneous electrical neuromodulation" or "percutaneous electrical neuromodulations" or "percutaneous neuromodulation therapies" or "percutaneous neuromodulation therapy" or "pet facilitated therapies" or "pet facilitated therapy" or "pet therapies" or "pet therapy" or "physical therapies" or "physical therapy" or "physical treatment" or "physio therapy" or "physiotherapy" or "pilates" or "plyometric drill" or "plyometric drills" or "plyometric exercise" or "plyometric exercises" or "plyometric training" or "plyometric trainings" or "plyometrics" or "postural drainage" or "proprioceptive neuromuscular facilitation (pnf) stretching" or "pulsed radio frequency treatment" or "pulsed radio frequency treatments" or "pulsed radiofrequency treatment" or "pulsed radiofrequency treatments" or "qi gong" or "qigong").tw,kf.

113. ("reflexology" or "rehabilitation exercise" or "rehabilitation exercises" or "relaxed stretching" or "remedial exercise" or "remedial exercises" or "resistance exercise" or "resistance exercises" or "resistance training" or "respiration exercise" or "respiration exercises" or "respiration therapies" or "respiration therapy" or "respiratory exercise " or "respiratory exercises" or "rolfing" or "shiatsu" or "shiatzu" or "shock wave therapies" or "shock wave therapy" or "shockwave therapies" or "shockwave therapy" or "shockwave treatment" or "shockwave treatments" or "soft tissue therapies" or "soft tissue therapy" or "spinal cord stimulation" or "spinal cord stimulations" or "spinal manipulation" or "spinal manipulations" or "spinal manipulative procedure" or "spinal manipulative procedures" or "spinal manipulative technique" or "spinal manipulative techniques" or "spinal stimulation" or "spinal stimulations" or "static exercise" or "static stretching" or "strength training" or "strength training" or "stretch shortening cycle exercise" or "stretch shortening cycle exercises" or "stretch shortening drill" or

"stretch shortening drills" or "stretch shortening exercise" or "stretch shortening exercises" or "stretching exercise" or "stretching exercises").tw,kf.

114. ("tai chi" or "t'ai chi" or "tai ji" or "taiji" or "taijiquan" or "therapeutic exercise" or "therapeutic exercises" or "therapeutic irrigation" or "therapeutic irrigations" or "transcutaneous electric nerve stimulation*" or "transcutaneous electric stimulation*" or "transcutaneous electrical nerve stimulation*" or "transcutaneous electrical stimulation*" or "transcutaneous electrostimulation*" or "transcutaneous nerve stimulation*" or "transdermal electrostimulation*" or "tui na" or "water immersion therapies" or "water immersion therapy" or "weight bearing exercise" or "weight bearing exercise program*" or "weight bearing exercises" or "weight bearing strengthening program*" or "weight lifting exercise program*" or "weight lifting strengthening program*" or "whirlpool bath" or "whirlpool baths" or "yoga" or "yoga" or "yogic meditation" or "zhi ya" or "zone therapies" or "zone therapy").tw,kf.

115. "prostheses and implants"/ or absorbable implants/ or artificial limbs/ or auditory brain stem implants/ or bioprosthesis/ or blood vessel prosthesis/ or bone-implant interface/ or breast implants/ or cochlear implants/ or exp dental implants/ or exp dental prosthesis/ or exp electrodes, implanted/ or exp embolic protection devices/ or eye, artificial/ or exp glaucoma drainage implants/ or exp heart, artificial/ or heart valve prosthesis/ or implants, experimental/ or exp internal fixators/ or exp joint prosthesis/ or larynx, artificial/ or exp lenses, intraocular/ or exp maxillofacial prosthesis/ or orbital implants/ or ossicular prosthesis/ or penile prosthesis/ or punctal plugs/ or septal occluder device/ or exp stents/ or suburethral slings/ or tissue expansion devices/ or tissue scaffolds/ or urinary sphincter, artificial/ or visual prosthesis/

116. ("accommodating intraocular lense" or "accommodating intraocular lenses" or "accommodating iol" or "accommodating iols" or "amalgam capsule" or "amplatzer occluder" or "amplatzer occluders" or "aqueous humor shunt" or "aqueous humor shunts" or "aqueous shunt" or "aqueous shunts" or "artificial arm" or "artificial arms" or "artificial cardiac valve" or "artificial extremities" or "artificial extremity" or "artificial eye" or "artificial eyes" or "artificial genitourinary sphincter" or "artificial genitourinary sphincters" or "artificial heart" or "artificial hearts" or "artificial larynges" or "artificial larynx" or "artificial leg" or "artificial legs" or "artificial limb" or "artificial limbs" or "artificial nose" or "artificial penis" or "artificial shoulder joint" or "artificial shoulder joints" or "artificial teeth" or "artificial tooth" or "artificial urinary sphincter" or "artificial urinary sphincters" or "artificial ventricle" or "artificial ventricles" or "ats valve").tw,kf.

117. ("bionic eye" or "bionic eyes" or "bioprostheses" or "bioprosthesis" or "bone anchor" or "bone anchors" or "bone nail" or "bone nails" or "bone pin" or "bone pins" or "bone plate" or "bone plates" or "bone screw" or "bone screws" or "bone wire" or "bone wires" or "breast endoprosthesis" or "carbomedics prosthetic heart valve" or "cardiac prosthetic valve" or "cardioseal occluder" or "cardioseal occluders" or "carpentier edwards bioprosthesis" or "cranioplasty plate" or "cranioplasty plate fastener" or "cranioplasty plate screw" or "crown" or "crowns" or "dental abutment" or "dental abutments" or "dental anchor" or "dental bridge" or "dental clasp" or "dental clasps" or "dental dowel" or "dental dowels" or "dental filling" or "dental fillings" or "dental laminate" or "dental laminates" or "dental onlay" or "dental onlays" or "dental permanent filling" or "dental permanent fillings" or "dental pin" or "dental restoration" or "dental restorations" or "dental slotted attachment" or "dental slotted attachments" or

"dental veneer" or "dental veneers" or "denture" or "dentures" or "disk valve" or "drug eluting stent" or "drug eluting stents").tw,kf.

118. ("electrolarynx" or "emboli protection devices" or "embolic protection device" or "embolic protection devices" or "embolic protection filter" or "embolic protection filters" or "embolism protection device" or "embolism protection devices" or "endoprosthesis oesophagei" or "endoprosthesis oesophagus" or "esophageal endoprosthesis" or "esophagus endoprosthesis" or "esophagus replacement" or "esophagus substitute" or "false teeth" or "fixed bridge" or "fixed bridges" or "glutaraldehyde stabilized graft" or "glutaraldehyde stabilized grafts" or "gynecological and obstetric implant" or "heart assist device" or "heart assist devices" or "heart assist pump" or "heart assist pumps" or "heart prosthetic valve" or "hydraulic artificial sphincter" or "implantable cardioverter defibrillator" or "implantable cardioverter defibrillators" or "implantable contact lens" or "implantable defibrillator" or "implantable defibrillators" or "implantable electrode" or "implantable electrodes" or "implantable neurostimulator" or "implantable neurostimulators" or "implantable stimulation electrode" or "implantable stimulation electrodes" or "implanted electrode" or "implanted electrodes").tw,kf.

119. ("implanted nerve stimulation electrodes" or "implanted neurostimulator" or "implanted neurostimulators" or "implanted stimulation electrode" or "implanted stimulation electrodes" or "implants" or "inlays" or "internal fixation device" or "internal fixation devices" or "internal fixator" or "internal fixators" or "intracoronal attachment" or "intracoronal attachments" or "intraocular lens" or "intraocular lenses" or "keratoprosthesis" or "kirschner wires" or "krupin valves" or "male sling" or "male slings" or "mamma endoprosthesis" or "mammary endoprosthesis" or "maryland bridge" or "mechanical heart" or "mechanical heart valve" or "mesh sling" or "mesh slings" or "midurethral sling" or "midurethral slings" or "midurethral tape" or "midurethral tapes" or "molteno shunts" or "mosaic bioprosthesis" or "multifocal intraocular lense" or "multifocal intraocular lenses" or "multifocal iol" or "multifocal iols").tw,kf.

120. ("obturator" or "ocular cosmetic shell" or "ocular peg" or "oesophageal endoprosthesis" or "oesophagus endoprosthesis" or "oesophagus replacement" or "overdenture" or "overdentures" or "pedicle screw" or "pedicle screws" or "percutaneous aortic valve" or "percutaneous aortic valves" or "plastic tooth" or "porcelain tooth" or "post and core technic" or "post and core technique" or "prostheses" or "prosthesis" or "prosthetic device" or "prosthetic system" or "prosthetic valve" or "pubovaginal sling" or "pubovaginal slings" or "punctal plug" or "punctal plugs" or "resin bonded bridge" or "resin bonded bridges" or "scleral shell" or "septal occluder" or "septal occluders" or "stent" or "stents" or "suburethral sling" or "suburethral slingplasty" or "suburethral slings" or "suburethral tape" or "suburethral tapes" or "suprapubic arc sling" or "surgical mesh" or "suture anchor" or "suture anchors" or "synthetic vascular graft").tw,kf.

121. ("tension free sling" or "tension free slings" or "tension free transvaginal tape" or "tension free vaginal sling" or "tension free vaginal slings" or "tension free vaginal tape" or "tension free vaginal tapes" or "tensionless vaginal tape" or "tensionless vaginal tapes" or "tips endoprosthesis" or "tissue engineered vascular graft" or "tissue engineered vascular grafts" or "tissue expander" or "tissue expanders" or "tissue expansion device" or "tissue expansion devices" or "tissue scaffold" or "tissue scaffolding" or "tissue scaffoldings" or "tissue scaffolds" or "tooth bridge" or "tot sling" or "tot slings" or "tot technique" or "transabdominal mesh" or

"transjugular intrahepatic portosystemic shunt endoprosthesis" or "transobturator sling" or "transobturator slings" or "transobturator tape" or "transobturator tapes" or "transvaginal slings" or "tvt sling" or "tvt slings" or "umbrella filter" or "umbrella filters" or "urethral sling" or "urethral slings" or "vascular assist device" or "vascular assist devices" or "vena cava filter" or "vena cava filters" or "viatorr" or "voice button").tw,kf.

122. rehabilitation/ or "activities of daily living"/ or art therapy/ or bibliotherapy/ or cardiac rehabilitation/ or "correction of hearing impairment"/ or communication methods, total/ or lipreading/ or manual communication/ or sign language/ or early ambulation/ or music therapy/ or neurological rehabilitation/ or stroke rehabilitation/ or occupational therapy/ or recreation therapy/ or "rehabilitation of speech and language disorders"/ or language therapy/ or myofunctional therapy/ or speech, alaryngeal/ or speech, esophageal/ or speech therapy/ or voice training/ or rehabilitation, vocational/ or telerehabilitation/

123. Alcoholics Anonymous/

124. Substance Abuse Treatment Centers/

125. Opiate Substitution Treatment/

126. ("aa meetings" or "accelerated ambulation" or "activities of daily living" or "alaryngeal speech" or "alaryngeal voice" or "alaryngeal voice production" or "alaryngeal voice productions" or "alcoholic anonymus" or "art therapies" or "art therapy" or "art treatment" or "auditory perception correction" or "auditory perception corrections" or "augmentative communication" or "bibliotherapies" or "bibliotherapy" or "bladder training" or "cheirology" or "chronic limitation of activity" or "community reintegration" or "constraint induced therapy" or "correction of auditory perception" or "correction of hearing impairment" or "correction of hearing loss" or "dactylology" or "dactylophasia" or "daily living activities" or "daily living activity" or "drug abuse treatment" or "drug abuse treatment centers" or "drug dehabituation" or "drug treatment center" or "drug treatment centers" or "early ambulation" or "early mobilisation" or "early mobilization" or "ergotherapy" or "esophageal speech" or "esophageal speechs" or "esophageal voice" or "esophagus voice" or "functional assessment" or "functional readaptation" or "functional training" or "habilitation" or "hearing impairment correction" or "hearing impairment corrections" or "hearing loss correction" or "hearing loss corrections" or "language therapies" or "language therapy" or "language training" or "language trainings" or "lip reading" or "lip readings" or "lipreading" or "lipreadings" or "logopedic education" or "logopedic training").tw,kf.

127. ("manual communication" or "manual communications" or "methadone maintenance" or "methadone treatment" or "moblization" or "music therapy" or "myofunctional therapies" or "myofunctional therapy" or "na meetings" or "narcotics anonymous" or "neurorehabilitation" or "nicotine replacement therapy" or "non laryngeal speech" or "non laryngeal voice" or "nonlaryngeal speech" or "nonlaryngeal voice" or "occupation therapy" or "occupational therapies" or "occupational therapy" or "oesophageal speech" or "oesophageal voice" or "oesophagus speech" or "oesophagus voice" or "opiate replacement therapies" or "opiate replacement therapy" or "opiate replacement treatment" or "opiate substitution therapy" or "opiate substitution treatment" or "opiate substitution treatments" or "opioid replacement therapies" or "opioid replacement therapy" or "opioid replacement treatment" or "opioid

substitution therapies" or "opioid substitution therapy" or "opioid substitution treatment" or "opioid substitution treatments" or "oral myotherapies" or "oral myotherapy" or "orofacial myologies" or "orofacial myology" or "orofacial myotherapies" or "orofacial myotherapy" or "readaption" or "readjustment" or "recreation therapies" or "recreation therapy" or "recreational therapies" or "recreational therapy" or "rehabilitation" or "rehabilitative treatment" or "rejuvenation" or "resocialization" or "revalidation" or "sensori motor integration" or "sensorimotor integration" or "sign language" or "sign languages" or "smoking cessation program" or "smoking cessation programme" or "sociotherapy" or "speech education" or "speech reeducation" or "speech therapies" or "speech therapy" or "speech training" or "speechreading" or "speechreadings" or "stroke rehab" or "substance abuse treatment centers" or "telerehabilitation" or "telerehabilitations" or "total communication" or "vocal reeducation" or "vocational retraining" or "voice training" or "voice trainings").tw,kf.

128. Spina Bifida Cystica/

129. ("myelodysplasia (spinal dysraphism)" or "neuroschisis" or "open spina bifida" or "spina bifida aperta" or "spina bifida cystica" or "spina bifida manifesta" or "spinal dysraphia" or "spinal dysraphic state" or "spinal dysraphic states" or "spine dysgraphia").tw,kf.

130. Spina Bifida Occulta/

131. ("closed spinal bifida" or "dermal sinus" or "occult spina bifida" or "spina bifida occulta" or "occult spinal dysraphia").tw,kf.

132. Wheelchairs/

133. ("wheel chair" or "wheel chairs" or wheelchair or wheelchairs).tw,kf.

134. or/62-133

135. 61 and 134

136. 135 not 2018*.ed. [pre 2018 records]

137. 135 and (201801* or 201802* or 201803*).ed. [records for Jan, Feb, Mar 2018]

138. 136 or 137 [records to Mar 31, 2018]

~ ~ ~ End of Appendix ~ ~ ~
